# The Impacts of Obesity and Metabolic Abnormality on Carotid Intima-Media Thickness and Non-Alcoholic Fatty Liver Disease in Children from an Inland Chinese City

**DOI:** 10.3390/jcm3010323

**Published:** 2014-03-20

**Authors:** Xiao-Yue Wang, Xiang-Hua Zhang, Chao Hua Yao, Hong-Hui Zhu, Liang Zhang

**Affiliations:** 1 Departments of Endocrinology, the First People’s Hospital, 39 Dongmaoling Road, Yueyang 414000, Hunan, China; E-Mails: 2050wxy@sina.cn (X.-Y.W.); xianghua8191@163.com (X.-H.Z.); 2 Palmer Laboratory of Cell & Molecular Biology, PCCR, Palmer College of Chiropractic-Florida, 4705 S. Clyde Morris Blvd, Port Orange, FL 32129, USA; E-Mail: chao.yao@palmer.edu; 3 Departments of Ultrasound, the First People’s Hospital, 39 Dongmaoling Road, Yueyang 414000, Hunan, China; E-Mail: zhh8996@163.com

**Keywords:** Yueyang, metabolic syndrome, carotid intima-media thickness, public health, non-alcoholic fatty liver disease

## Abstract

The Chinese inland, where low child obesity and overweight rates were reported in earlier studies, has recently experienced rapid economy changes. This may impact children’s health. In the present study, we investigated the obesity rate, metabolic health status, and their impacts on carotid intima-media thickness (IMT) and non-alcoholic fatty liver disease (NAFLD) among children from Yueyang, an inland city of China. We found that the obesity rate was about 5% for both 7- and 11-year olds. Overweightness rates were 9.5% and 11.5% for the 7- and 11-year olds, respectively. Clinical and laboratory examinations revealed significant differences among different weight groups in the 11-year old volunteers, which were absent in the 7-year olds. Further statistical analysis showed that: age, BMI, blood pressure, triglyceride level, and metabolic abnormality were positively correlated to carotid IMT; triglyceride level, obesity, male, and the number of metabolic abnormalities were independent risk factors for NAFLD in these children. Our study suggests that: childhood overweightness and obesity are now epidemic in Yueyang, which have contributed to increased carotid IMT and may also increased NAFLD incidents; and serum triglyceride level is a critical factor in the development of childhood NAFLD. Thus, childhood metabolic health warrants further vigorous research in the inland of China.

## 1. Introduction

Economic development impacts every aspect of human life including public health. Since implementing the “Reform and Open-up” policy in China, in 1979, the country has changed tremendously, particularly on the economic front. The Chinese economic boom first occurred in the coast cities and then in big cities during the 1990s, which led to improvements of living standards among the people. Such improvements often accompany changes of life-style and dietary habit (particularly increased meat consumption), which, in turn, unleashes a great impact on public health. Indeed, studies reported a rising prevalence of obesity and being overweight among young children and adolescents from 1985 to 2000 in Beijing and the coast areas, which was absent in the less developed inland cities [1,2]. However, since joining the WTO (World Trade Organization) in 2001, the economic boom has spread even deeper into the vast Chinese inland, where a majority of Chinese resides but no current data are available regarding their children’s health status.

Particularly, there is scarce information regarding child metabolic health in China [3]. We are interested in children’s metabolic health conditions in the Chinese inland, which would provide forward predictions on certain potential future health problems. In addition these problems could be addressed in our current preventive care. Metabolic syndrome is one of the most common metabolic disorders, including obesity, hypertension, dyslipidemia, insulin resistance (IR), and type 2 diabetes. Metabolic syndrome associates with adverse cardiovascular outcomes, which can be traced back to early vascular changes in young children and adolescents with the condition [4]. It is well known that dyslipidemia causes artery impairments and even atherosclerosis [5,6]. Lipid deposition in artery intima may start during early childhood, which accumulate and eventually lead to serious pathological damages during adulthood [7,8]. Increased carotid intima-media thickness (IMT) is positively correlated to coronary artery disease in adults [9,10]. Researchers in the pediatrics field also measure carotid IMT to monitor children’s health [6,8]. Non-alcoholic fatty liver disease (NAFLD) is also a common complication of metabolic syndrome [11,12], with an average global prevalence about 20% [13]. However, NAFLD prevalence in children has been rarely studied.

In the present study, we set out to determine the prevalence of childhood overweightness, obesity, and metabolic syndrome, and their relationships to carotid IMT and NAFLD, in Yueyang city of Hunan Province, a mid-size city in China’s inland. This study may provide a window to understand childhood obesity and metabolic problems in this vast area.

## 2. Methods

### 2.1. Subjects and Study Designing

From September to November of 2006, 2124 7-year old and 1884 11-year old students from 5 randomly selected elementary schools in Yueyang city were screened for overweight and obesity using the standard BMI (body mass index) protocol (We chose these two age groups to control study cost while still maintain a significant age difference). Height (Ht), weight (Wt), waist circumference (WC), hip circumference (HC) were measured for all subjects. BMI (kg/m^2^) was calculated by Wt/Ht^2^. Three weight groups were then classified by comparing each individual BMI value to the average BMI value of the same sex and age population obtained in the current study: overweight group, BMI ≥ 85% of the average; obesity group, BMI ≥ 95% of the average; the rest belong to the normal group. Then, 206 pupil volunteers (randomly selected from all those participated in the initial screening) from different weight groups were further examined for blood pressure (BP), fasting plasma glucose (FPG), lipid-profile including high density cholesterol (HDL-C), low density cholesterol (LDL-C), cholesterol (CHOL), triglyceride (TG), plus biochemical analysis on transaminase activities, such as alanine aminotransferase (ALT) and aspartate aminotransferase (AST). According to their laboratory test results, these volunteers were further classified into 4 metabolic sub-groups, based on the guidelines (see below in diagnosis of metabolic syndrome) from International Diabetes Foundation on diagnosis of metabolic syndrome (MS) in children and adolescents [14]: MS (≥3 abnormalities), M2 (2 abnormalities), M1 (1 abnormality), M0 (no abnormality). Meanwhile, a SIEMENS G60S ultrasound machine was used to measure carotid IMTs and obtain liver imagines from the volunteers. The studying protocol was pre-approved by the Research Ethics Committee of the First People’s Hospital in Yueyang City. Parent consents from all participating children were obtained prior to their participation in this study.

### 2.2. Diagnosis of Metabolic Syndrome

We used the following guidelines to diagnose metabolic syndrome [14]: For children from 10 to <16 years old, a positive diagnosis was made if an individual was presented with abdominal obesity plus any two or more of abnormalities: (1) abdominal obesity: WC ≥ 90% of the average; (2) blood pressure: Systolic ≥ 130 mmHg or diastolic ≥ 85 mmHg; (3) HDL-C < 1.03 mmol/L (40 mg/dL); (4) TG: ≥ 1.7 mmol/L (150 mg/dL); (5) glucose ≥ 5.6 mmol/L (100 mg/dL), or known as T2DM (type 2 diabetes mellitus), or FPG (fasting plasma glucose) ≥ 5.6 mmol/L, and positive on a further oral glucose tolerance test (OGTT).

For children <10 years old, metabolic syndrome could not be diagnosed. However, further measurements were made if there was a family history of metabolic syndrome, T2DM, dyslipidaemia, cardiovascular disease, hypertension and/or obesity, and the numbers of metabolic abnormalities were recorded for these children.

### 2.3. Diagnosis of NAFLD

We followed the guidelines to diagnose NAFLD (except liver biopsy was not performed due to the associated high cost) published by Angulo [13]: (1) No drink or average daily consumption less than 40 g ethanol (less than 20 g in females); (2) Excluded the exogenous factors, such as drugs, poison, infection, such as hepatitis B or C; (3) Liver ultrasound image appeared of fatty suffusion; (4) Patients with existing risk factors for metabolic disorders had slightly elevated levels of fasting ALT (>28 U/L for boys and 24 U/L for girls); (5) Patients had risk factors, such as rapid weight gain, central obesity, impaired fasting plasma glucose, dyslipidemia, and hypertension.

An individual would be diagnosed as NAFLD: If he or she satisfied the first, second, and any one of the third and fourth; or the fifth together with the third and/or fourth being improved after changing lifestyle. (Determination of liver fatty suffusion: Sonography was conducted by a qualified radiologist using a SIEMENS G60S ultrasound machine. Increased reflectivity of the hepatic parenchyma on conventional plane images was classified as positive for liver fatty suffusion).

### 2.4. Statistical Analysis

The SPSS 11.5 package (SPSS Inc., Chicago, IL, USA) was used for statistical analysis. Student’s *t*-test was used to compare two groups after homoscedasticity test; analysis of variance (ANOVA) and analysis of covariance (COANOVA) were used to compare multiple groups. For comparison between proportions, the *χ*^2^ test was used. Pearson analysis and logistical regression were used to evaluate the correlation and relationship between different factors. *p* < 0.05 was deemed significant.

## 3. Results

### 3.1. The Prevalence of Overweight/Obesity in Children Living in Yueyang City

For initial screening, we studied a total of 4008 students—Among them 2180 males (54.4%) and 1828 female (45.6%) with an average BMI of 16.82 ± 2.69 (kg/m^2^). Table 1 shows the prevalent rates of being overweight and obesity among different age and sex groups were calculated. Comparing the prevalence of overweight/obesity between different sex groups, we obtained a *χ*^2^ value of 93.00 (*p* < 0.001), indicating the prevalence of overweight/obesity in males was higher than in females.

### 3.2. The Age Effect on Clinical Features in Different Weight Groups

After initial screening, 206 volunteers from different weight groups were randomly selected for further detailed clinical tests. Table 2 summarizes the detailed results of the clinical tests on 97 7-year old and 109 11-year old volunteer pupils—Among them 114 males and 92 females. In the 7-year old children, BMI, WC, and HC were significantly different among different weight groups, while SBP (systolic blood pressure), DBP (diastolic), PP (pulse pressure), TG, HDL-C, and FPG showed no significant difference. In the 11-year olds, BMI, WC, HC, SBP, DBP, PP, and TG were all significantly different among different weight groups. However, no significant difference was found in HDL-C or FPG among different weight groups in the 11-year olds.

**Table 1:** Prevalence rates of being overweight and obesity in 7- and 11-year old Chinese children of Yueyang city.

Group	Normal	Overweight	Obese
7-year male (1105)	891 (80.6) ^#^	133 (12.0) ^#^	81 (7.3) ^#^
7-year female (919)	828 (90.1)	60 (6.5)	31 (3.4)
7-year total (2024)	1719 (84.9)	193 (9.5)	112 (5.5)
11-year male (1075)	834 (90.3) *	163 (15.2) *	78 (7.3) *
11-year female (909)	821 (90.3)	66 (7.3)	22 (2.4)
11-year total (1784)	1655 (83.4)	229 (11.5)	100 (5.0)

^#^ Compared with 7-year old female, the prevalence of normal, being overweight, and obese *p* < 0.001; * Compared with 11-year old female, the prevalence of normal, being overweight, and obese *p* < 0.001.

**Table 2:** Clinical features in different weight groups of 7- and 11-year old volunteers.

Age	Wt	*n* (m/f)	BMI (kg/m^2^)	WC (cm)	HC (cm)	SBP (mmHg)	DBP (mmHg)
**7years**	N	17/14	16.5 ± 0.5 *^,^^★^	52.3 ± 1.8 *^,^^★^	63.7 ± 4.4 ^★^	97.2 ± 2.5	64.1 ± 2.1
	O	17/16	18.4 ± 0.5 ^☆^^,^^★^	58.2 ± 2.4 ^☆^^,^^★^	64.5 ± 4.6 ^★^	99.5 ± 4.2	64.7 ± 3.2
	F	20/14	21.3 ± 1.2 ^☆^^,^*	65.4 ± 3.6 ^☆^^,^*	70.1 ± 4.0 ^☆^^,^*	100.0 ± 6.9	65.2 ± 2.4
**11 years**	N	17/17	17.2 ± 1.7 *^,^^★^	60.4 ± 4.4 *^,^^★^	73.3 ± 5.8 *^,^^★^	101.0 ± 9.6 *^,^^★^	66.4 ± 5.1 ^★^
	O	20/14	22.1 ± 0.8 ^☆^^,^^★^	70.7 ± 4.1 ^☆^^,^^★^	79.5 ± 5.4 ^☆^^,^^★^	107.1 ± 7.0 ^☆^^,^^★^	68.4 ± 6.6 ^★^
	F	23/17	25.8 ± 2.3 *^,^^☆^	77.9 ± 6.4 ^☆,^*	83.0 ± 6.4 ^☆,^*	116.6 ± 9.5 ^☆,^*	74.9 ± 7.7 ^☆,^*
**Age**	**Wt**	**PP** **(mmHg)**	**TG (mmol/L)**	**HDL-C (mmol/L)**	**FPG (mmol/L)**	**FINS **	**HOMA-IR**
**7 years**	N	33.0 ± 2.3	0.81 ± 0.20	1.39 ± 0.16	4.88 ± 0.35	2.25 ± 0.22	0.72 ± 0.26
	O	34.8 ± 3.9	0.85 ± 0.18	1.29 ± 0.23	4.85 ± 0.28	2.20 ± 0.16	0.66 ± 0.18
	F	34.8 ± 5.9	0.87 ± 0.23	1.34 ± 0.18	4.83 ± 0.42	2.37 ± 0.34	0.83 ± 0.36
**11 years**	N	34.6 ± 6.3 *^,^^★^	0.79 ± 0.24 *^,^^★^	1.33 ± 0.21	4.90 ± 0.35	2.13 ± 0.23 *^,^^★^	0.61 ± 0.24 *^,^^★^
	O	38.8 ± 6.4 ^☆^	1.05 ± 0.40 ^☆^	1.32 ± 0.19	4.83 ± 0.44	2.44 ± 0.27 ^☆^^,^^★^	0.89 ± 0.28 ^☆^^,^^★^
	F	41.7 ± 6.4 ^☆^	1.21 ± 0.51 ^☆^	1.23 ± 0.19	4.81 ± 0.23	2.74 ± 0.34 *^,^^☆^	1.20 ± 0.36 *^,^^☆^

N: normal; O: overweight; F: obese. All numbers are expressed as mean ± SD. ^☆^: compared to normal group, *p* < 0.05; *: compared to overweight group, *p* < 0.05; ^★^: compared to obese group, *p* < 0.05; BMI: body mass index; BP: blood pressure; DBP: diastolic; BP: FINS, fasting insulin; FPG: fasting plasma glucose; HC: hip circumference; Ht: Height; HDL-C: high density cholesterol; HOMA-IR: insulin resistance index; PP: pulse pressure; SBP: systolic blood pressure; TG: triglyceride; Wt, weight; WC: waist circumference.

### 3.3. Carotid IMT Correlated to Metabolic Status in Children

There was a general trend that the male pupils had thicker carotid IMT than the females in the study. However, this sex difference was only statistically significant in the 11-year old children (boys 0.466 ± 0.066, girls 0.46 ± 0.061; *p* < 0.05). Even though there was no MS sub-group in 7-year old children, variance analysis on IMT in other different metabolic sub-groups revealed no significant difference (*p* = 0.084) in children of this age group. However, Student’s *t* test did show that the IMT in sub-group M2 was slightly and significantly (*p* = 0.028) thicker than in sub-group M0 (the normal) in the 7-year olds. Variance analysis on IMT among different metabolic sub-groups from the 11-year old children revealed a significant difference (*p* = 0.034). Among the 11-year olds, the IMT in MS sub-group was thicker than that in the sub-group M0 or M1 (*p* = 0.004 or 0.030, respectively). These data indicate that the carotid IMT thickens rapidly as metabolic conditions become worse in children.

Table 3 shows the relationship between IMT and various clinical features by Pearson analysis. Age, BMI, WC, HC, SBP, DBP, PP, and TG were all positively correlated to IMT (*p* < 0.05); metabolic abnormality was also positively correlated to IMT (*p* = 0.004). However, HDL-C and FPG had no significant correlation with IMT (*p* = 0.264 and 0.643, respectively).

**Table 3:** Relationship between clinical features and carotid intima-media thickness (IMT) in Children from Yueyang.

Age		BMI (kg/m^2^)	WC (cm)	HC (cm)	SBP (mmHg)	DBP (mmHg)	TG (mmol/L)	HDL (mmol/L)	FPG (mmol/L)	MS
*r*^2^	0.370	0.487	0.484	0.485	0.373	0.312	0.232	−0.116	0.048	0.328
*p*	0.001	0.001	0.001	0.001	0.001	0.002	0.025	0.458	0.643	0.001

After collecting detailed clinic information from 206 volunteers, Pearson analysis was used to determine the relationship between each clinical feature and carotid IMT (intima-media thickness). BMI: body mass index; BP, blood pressure; DBP: diastolic BP; FPG, fasting plasma glucose; HC: hip circumference; Ht: Height; HDL-C: high density cholesterol; MS: metabolic syndrome; SBP: systolic blood pressure; TG: triglyceride; WC: waist circumference.

### 3.4. NAFLD Prevalence and Risk Factors

We found the overall prevalence of NAFLD was about 7.7% in the 7-year old children studied and detected no significant difference among different BMI or metabolic groups (*p* = 0.638). However, we did detect significant differences in NAFLD prevalence among different groups in the 11-year old children (*p* = 0.006). When grouped by BMI, the prevalence of NAFLD in the obese group of 11-year olds was 31.11%, which was much higher than the normal group (*p* < 0.001). When compared by their metabolic status for the 11-year old children, we found that the prevalence of NAFLD in MS subgroup was significant higher than M0 subgroup (50.0% *vs.* 8.0%, *p* = 0.001). Using NAFLD prevalence as a dependent variable and other clinical features as independent variables in a logistic-regression analysis, we found that TG levels, obesity, male, and the number of metabolic abnormalities were positively related to NAFLD (Table 4). As shown in Table 4, the chance to have NAFLD increases as the TG level arises: when TG level arises 1 mmol/L, the chance to have NAFLD increases 13 times. Meanwhile, obese children have 5.4 fold more chances to get NAFLD than normal children; males are 4.8 times more likely than females to have NAFLD. Finally, the number of metabolic abnormalities increases by one, the chance of having NAFLD increases by 2.4 fold (Table 4).

**Table 4:** Logistic-regression analysis of different parameters on non-alcoholic fatty liver disease (NAFLD) incidents.

Variable	*b*	S.E.	Wald	*p*	OR	95% CI
X_2_	1.564	0.661	5.600	0.018	4.779	1.308–17.458
X_3_	1.687	0.729	5.354	0.021	5.402	1.294–22.543
X_4_	0.878	0.409	4.617	0.032	2.406	1.294–22.543
X_12_	2.569	0.892	8.296	0.004	13.058	2.273–75.024
Constant	−7.587					

Logistic-regression was used to analyze the impact of each clinical variable on NAFLD incidents among the volunteers. Several significant variables are identified and listed here: X_2_, sex; X_3_, obesity; X_4_, the number of metabolic abnormality; X_12_, triglyceride level. Abbreviations: OR, odds ratio; CI, confidence interval; *b*, co-efficient estimates; NAFLD, non-alcoholic fatty liver disease.

## 4. Discussion

This report represents the first attempt to study obesity and metabolic abnormalities in children living in a Chinese inland city since the country joined the WTO in 2001. The prevalence of childhood overweight/obesity in Yueyang city is currently high, and is getting closer to that of Beijing in 2000 reported by Ji *et al.* [1,2]. This finding indicates that recent economic advancement in the Chinese inland may have already impacted the health of children living in this vast area as evident by their increased obesity and overweight rates. Our findings showed trends similar to some previous studies in different locations: the prevalence of overweight/obese in boys is higher than in girls [15]; and carotid IMTs in obese children were thicker than in normal children [16]. In addition, for the first time, we have identified several important risk factors that contribute to the development of NAFLD in children. Among them, serum TG level is of particularly importance.

Carotid IMT is a faithful indicator to evaluate cardiovascular health status [4,10]. Currently using high frequency ultrasound to measure carotid IMT is the best approach to detect early atherosclerosis, as ultrasound is more accurate in measuring artery thickness than Doppler ultrasound or angiography [10]. Previous studies reported that patients with metabolic syndrome showed significant difference in arterial compliance and artery IMT [17,18]. Zhu *et al*. [16] found that IMTs in obese children were thicker than in normal children. Our study further showed that the IMT was positively correlated to age, BMI, WC, HC, SBP, DBP, PP, and TG. We also found that there was no correlation between HDL-C and IMT in children, which, however, are negatively correlated in adults [19]. This may be due to the young age of the subjects studied and/or the low numbers, particularly the limited cases of metabolic syndrome in children. No correlation was detected between FPG and IMT in the present study, which is in agreement with a previous study reported from Germany [20]. The aggregation of metabolic abnormality was also found to be positively correlated to IMT in our study, which implicates multiple risk factors for vascular problem. As the number of risk factors increases, blood vessel impairments would gradually become more and more evident, particularly as patients grow older (see more discussion below). After adjusting diet and exercise, children’s vascular function can be greatly improved. Thus, metabolic abnormalities in children are usually reversible, and should be detected and treated rigorously as early as possible (mostly by changing lifestyle).

The other focus in our study was NAFLD—A problem also closely related to metabolic syndrome. The pathophysiological development of NAFLD includes initial lipid accumulation and mitochondrion impairment in liver cells, which may lead to cell death and result in non-alcoholic steatohepatitis and fibrosis (two hallmarks of NAFLD) [21]. In our study, the prevalence of NAFLD in obese children was significant higher, which is in agreement with other reports [22,23]. In addition, we found that as the number of metabolic abnormalities increase, the prevalence of NAFLD is ascending. This phenomenon was also observed by others [24]. More importantly, we are the first to show that TG and male are new independent risk factors for NAFLD in Chinese children. Particularly, increased serum TG level dramatically promoted NAFLD incidents (Table 4). Therefore, it seems we should pay more attention to children who have high serum TG levels, especially to male Chinese children. These results are consistent with the well-known Chinese culture bias on overfeeding male children.

Age is also an important factor in metabolic abnormality. We have detected different metabolic abnormality profiles in two different age groups of children (Table 2) and found that metabolic abnormality is a risk factor for both carotid IMT thickening and NAFLD incidents. Though in this snap-shot study we have not investigated how long obesity will last, obesity is a long standing feature unless the individual changes his or her life style. Thus, dyslipidemia would become more pronounced as the children grow older. Various lipids especially cholesterol, would be deposited in artery intima, and induce their thickening, which would lead to confined vessels and increased resistance at acroteric-vessles. These would enhance the pathophysiological development of numerous cardiovascular disorders such as atherosclerosis and hypertension [7]. In addition, excessive visceral fat mass was reported to have close relationship with metabolic abnormality and insulin resistance [25,26]. Thus, glucose metabolic abnormality could also be developed when obese children grow older. As mentioned above, metabolic abnormities are usually reversible in children via changing lifestyles. It is also worthy to note that physical activities may modify the evolution of NAFLD in adults [27]. Finally, in the literature, there are controversial reports regarding the NAFLD-IMT association in adults [28,29]. A recent report indicated that, NAFLD severity did not correlated with increased IMT in adult patients with normal or elevated gamma-glutamyltransferase activity [30]. While we have not studied this relationship in our study, it would be interesting to explore the difference between children and adults in that regard. These data suggest a complex relationship between NAFLD and IMT involving numerous factors.

## 5. Conclusions

Childhood overweight and obesity in Yueyang city are epidemic now, accompanying with various metabolic abnormalities that contribute to increased carotid IMT and NAFLD incidents among the children. While detecting similar risk factors for carotid IMT reported by others, we, for the first time, have identified several important risk factors for NAFLD in children, of which increased serum TG level is of critical importance. Thus, we cannot ignore the negative side effects brought out with the development of a good economy. It is pressing now for the public in the vast Chinese inland area to act together in preventing childhood overweightness and obesity, which will greatly benefit combat against cardiovascular diseases, diabetes, and other metabolic disorders that are rising in the Chinese society.

## Abbreviations

ALT: alanine aminotransferase
ANOVA: analysis of variance
AST: aspartate aminotransferase
BMI: body mass index
BP: blood pressure
CHOL: cholesterol
COANOVA: analysis of covariance
DBP: diastolic BP
FPG: fasting plasma glucose
HC: hip circumference
Ht: Height
HDL-C: high density cholesterol
IR: insulin resistance
IMT: intima-media thickness
LDL-C: low density cholesterol
MS: metabolic syndrome
NAFLD: non-alcoholic fatty liver disease
OGTT: oral glucose tolerance test
PP: pulse pressure
SBP: systolic blood pressure
T2DM: type 2 diabetes mellitus
TG: triglyceride
Wt: weight
WC: waist circumference
